# Belatacept and the Risk of Cytomegalovirus, BK Polyomavirus, and Epstein–Barr Virus Post‐transplant Lymphoproliferative Disease After Kidney Transplantation: A Meta‐Analysis and Systematic Review

**DOI:** 10.1155/joot/3256272

**Published:** 2026-09-12

**Authors:** Cybele Lara R. Abad, Raymund R. Razonable

**Affiliations:** ^1^ Department of Medicine, Division of Infectious Diseases, Philippine General Hospital, University of the Philippines-Manila, Manila, Philippines, upm.edu.ph; ^2^ Department of Medicine, Division of Public Health, Infectious Diseases and Occupational Medicine, Rochester, Minnesota, USA, duhs.edu.pk; ^3^ The William J Von Liebig Center for Transplantation and Clinical Regeneration, Mayo Clinic College of Medicine and Sciences, Rochester, Minnesota, USA, mayoclinic.org

**Keywords:** belatacept, BK virus, costimulation blocker, cytomegalovirus, Epstein–Barr virus, kidney transplant, post-transplant lymphoproliferative disease, safety

## Abstract

**Background:**

The long‐term risk of cytomegalovirus (CMV), Epstein–Barr virus (EBV)‐associated post‐transplant lymphoproliferative disorder (PTLD), and BK viral infections from belatacept is unclear. This review aimed to evaluate these outcomes.

**Methods:**

Several databases were reviewed from inception through September 2025 using the keywords “belatacept” and “kidney transplant” or “safety” and “viral infection”. We included case reports, randomized controlled trials (RCTs), and nonrandomized trials (NRTs). Outcomes were prespecified according to dose (more‐intense or less‐intense), type of infection (CMV, EBV‐PTLD, or BK), and follow‐up period: short term (1–2 years), medium term (3–5 years), and long‐term (> 5 years). RCTs and NRTs were analyzed separately, and case reports were summarized. For binary outcomes, results were presented as risk ratios (RR), with 95% confidence intervals (CI). All statistical analyses were performed using Revman 5.2.

**Results:**

Sixty‐four studies (23 RCTs, 28 NRTs, and 13 case reports) were included. Of 23 RCTs, 11 were primary studies while the remainder were follow‐up reports of original RCTs. Most NRTs (16/28) included a comparator arm. In the meta‐analyses of RCTs only, the risks of CMV, EBV‐PTLD, and BK virus infection were similar between patients receiving belatacept and those receiving calcineurin inhibitors (CNIs). During medium‐term follow‐up for RCTs, BK virus infection was twice as likely among those receiving more‐intense compared to less‐intense belatacept (RR: 2.08 [CI 1.03, 4.20, *I* ^2^ = 0]). Among NRTs, the risk of CMV infection was two‐fold higher among those receiving belatacept versus CNI, with an RR of 2.15 [CI 1.58, 2.93, *I* ^2^ = 66%]; BK DNAemia or viremia was also greater than two‐fold higher, with an RR of 2.59 [CI 1.10–6.11].

**Conclusions:**

The risks of CMV and EBV‐PTLD after kidney transplantation were not significantly increased by belatacept based on RCTs, though a higher risk of BK polyomavirus infection was observed among patients receiving a more‐intense dose of belatacept beyond the second year. The risk of CMV and BK polyomavirus infections may also be increased with real‐world belatacept use. However, these observations should be interpreted with consideration for the inherent limitations of NRTs.

## 1. Introduction

Belatacept is a first‐in‐class selective costimulation blocker that was designed to avoid the nephrotoxic and other adverse effects of calcineurin inhibitors (CNIs) [1–5]. It was approved for clinical use by the U.S. Food and Drug Administration (FDA) in June 2011 on the basis of three randomized controlled trials (RCTs) which compared belatacept to cyclosporine, in a regimen that concomitantly included mycophenolate mofetil (MMF) and steroids. In all three studies, belatacept was noninferior to CNIs in preventing acute rejection after kidney transplantation [6–8].

The distinct mechanism of action of belatacept warrants a thorough assessment of its infection risk. For example, short‐term results of the BENEFIT and BENEFIT‐EXT studies highlighted an increased frequency of Epstein–Barr virus (EBV)‐driven post‐transplant lymphoproliferative disorder (PTLD) in the group treated with belatacept compared with cyclosporine [8, 9]. Accordingly, belatacept use was contraindicated for EBV‐seronegative transplant recipients and those at increased PTLD risk. Fifteen years since its U.S. FDA approval, numerous studies have reported on the outcomes of belatacept, including the risk of other viral infections, with inconsistent results. A prior meta‐analysis was also undertaken over a decade ago [10], but this focused mainly on kidney‐related outcomes rather than infections. This study aimed to evaluate specific infection outcomes by (1) synthesizing data from trials and cohort studies that compared belatacept with other immunosuppression regimens, and (2) reporting cases of infections among those on belatacept therapy.

## 2. Methods

### 2.1. Information Sources and Search Strategy

With the assistance of a professional librarian, we searched multiple databases (PUBMED, EMBASE, SCOPUS, and MEDLINE/OVID) and identified clinical trials and other reports involving the use of belatacept from inception to September 15, 2025. Search terms included the keywords “belatacept” and “kidney transplant” or “safety” and “viral infection”. The complete search strategy is provided in Supporting File 1. Article references were reviewed for additional relevant reports. Our search was limited to publications in English or those with an English translation.

### 2.2. Eligibility Criteria

All trials or cohorts reporting primarily on cytomegalovirus (CMV), EBV, or PTLD after belatacept use among kidney transplant recipients within the study period were eligible for study inclusion and screened by both authors (CLA and RRR). Data on BK virus‐ or polyomavirus‐associated nephropathy (PyVAN), when described, were also captured. Case reports that reported any infection were included. We excluded abstracts, pediatric cases, and use of belatacept in other nonkidney organ transplants. Reports for which clinical information was inadequate or lacking, could not be extracted in detail, or included mixed populations (e.g., predominantly pediatric or nontransplant) were excluded. Informed consent was not required as these reports have been previously reported.

### 2.3. Data Collection and Analysis

#### 2.3.1. Selection of Studies

Titles and abstracts resulting from the search, as described above, were screened by both authors, who independently assessed retrieved abstracts, and if necessary, the full texts of these studies to determine which studies met the inclusion criteria. Disagreement about inclusion was resolved by discussion. No artificial intelligence–generated content (AIGC) tools such as ChatGPT and large language models (LLMs) were used for study selection. Where more than one report of a study existed, we used the first complete (index) study publication as the primary data source for patient demographics and baseline characteristics. Follow‐up studies were included to report infection‐related outcomes over time.

#### 2.3.2. Data Extraction and Management

Data extraction was carried out independently by two authors using standardized data extraction forms. Primary outcomes included the occurrence of CMV infection (inclusive of CMV DNAemia, viremia, infection, or disease), EBV‐driven PTLD, and BK infection (inclusive of BK DNAemia or viremia), as defined by the authors of the studies included in this meta‐analysis and systematic review. Secondary outcomes included the occurrence of BK virus‐ or polyomavirus‐associated nephropathy (PyVAN). Data were entered into RevMan 5.2 and an Excel spreadsheet. Statistical analyses were performed using RevMan 5.2.

#### 2.3.3. Assessment of Risk of Bias in Included Studies

The following items were independently assessed by two authors using the risk‐of‐bias assessment tool [11].

#### 2.3.4. Measures of Treatment Effect

For binary outcomes, results were expressed as risk ratios (RR) with 95% confidence intervals (CIs).

#### 2.3.5. Dealing With Missing Data

Missing data were neither replaced nor imputed.

#### 2.3.6. Assessment of Heterogeneity

For binary outcomes, we presented results as RR with 95% CI, showing the proportional change in risk for the study or participant characteristic listed versus the reference range comparison. Tests of heterogeneity among pooled subgroup estimates were calculated using univariate random‐effects meta‐regression.

#### 2.3.7. Assessment of Reporting Biases

We were unable to quantitatively assess the potential existence of publication and small study biases through funnel plots [11] or Egger’s regression because each outcome had < 10 studies.

#### 2.3.8. Data Synthesis

We pooled data from individual randomized and nonrandomized studies separately, using a random‐effects model, because the random‐effects model provides a more conservative estimate of effect in the presence of known or unknown potential heterogeneity. Case reports were described separately.

#### 2.3.9. Subgroup Analysis and Investigation of Heterogeneity

Subgroup analyses of outcomes were prespecified according to belatacept dosage (e.g., more intense [MI] or less intense [LI]), type of infection (CMV, EBV/PTLD, BK), and period of follow up predefined as short term (within 2 years, when most of these viral infections are anticipated to occur), medium term (3–5 years, when the risk of these infections are lower overall, but is augmented in patients who are overimmunosuppressed), and long‐term (> 5 years, when the risk of infection is anticipated to be minimal). This was intended to avoid counting studies twice (e.g., for multiple reports falling into the same time period, the study with the longer follow‐up period was used).

For binary outcomes, we presented results as RR with 95% CI, showing the proportional change in risk for the study or participant characteristic listed versus the reference range comparison. Tests of heterogeneity among pooled subgroup estimates were calculated using univariate random‐effects meta‐regression.

## 3. Results

**FIGURE 1:**
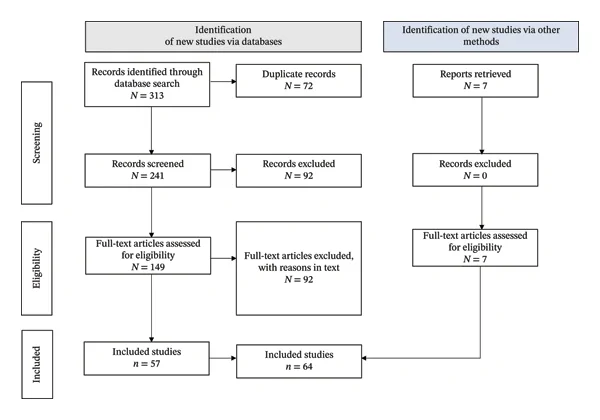
PRISMA flow diagram.

Our search identified a total of 313 studies across multiple databases (PUBMED *n* = 138, EMBASE *n* = 49, MEDLINE *n* = 126). After removal of duplicate reports and screening of titles and abstracts, 149 full‐text reports were assessed. An additional 92 reports were excluded after full‐text review for the following reasons: no infection outcomes (n=77), other infections of interest were assessed (n=7), infections occurred prior to belatacept use (n=1), studies used pre‐existing or duplicate data (n=5), pharmacokinetic‐dynamic studies (n=1), and immune suppression was mixed (n=1). After finding 7 more new studies, we included a total of 64 studies (Figure 1), of which 8 were Phase 2 studies [6, 12–18], 15 were RCTs [7, 8, 19–31], 28 were nonrandomized trials (NRTs) [32–59], and 13 were case reports [60–72].

### 3.1. All Selected Studies

#### 3.1.1. Study Characteristics

##### 3.1.1.1. Phase 2 Studies

A total of 540 study participants from 4 primary studies [6, 12, 15, 16] were included. Study populations ranged from 60 [16] to 218 [6] participants per study. All were multisite, multicountry studies, except for one [16]. There were 3 de novo studies [6, 12, 16], and one was a conversion study [15]. Of the remaining studies, one was a 5‐year long‐term extension study [17], and the other was a 10‐year follow‐up of the Vincenti 2005 study [18]. The rest were 2‐ and 5‐year follow‐up reports, respectively, of the Rostaing 2011 study [13, 14].

##### 3.1.1.2. Other Randomized Studies

A total of 2257 participants from 7 primary randomized studies [7, 8, 19, 20, 25, 26, 31] were included; the others were 2‐ [23, 24], 3‐ [27, 29], 5‐ [21, 28], or 7‐year [22, 30] follow‐up studies.

##### 3.1.1.3. Nonrandomized Trials

A total of 28 nonrandomized studies were included in this review [32–59]. The majority were retrospective cohorts (*n* = 15), some were case–control studies (*n* = 11), and a few were prospective cohorts (*n* = 2). The majority were published in the United States (*n* = 17) or Europe (*n* = 10; including France 7, Germany 2, and multiple European countries 1); 1 was from Argentina. Two retrospective studies were population‐based and used existing databases—the Scientific Registry of Transplant Recipients (SRTR) [33] and the Organ Procurement and Transplantation Network (OPTN) [39].

##### 3.1.1.4. Case Reports

Thirteen case reports were included [60–72]. The majority were from the United States (*n* = 7), followed by France (*n* = 4), Austria (*n* = 1), and Italy (*n* = 1). Most were male (9/13), and the median age was 54 (range 41–89) years. Of those that reported donor data (10/13), 7 were deceased donors. CMV status was mentioned only in 8 studies; of these, 2 were high‐risk mismatches (*D*+/R−), 4 were recipient seropositive, and 2 were recipient seronegative with unspecified donor status. Six were EBV recipient seropositive, and EBV status was not specified for the remainder. Many of these patients were converted to belatacept, with a median time to conversion of 3 (range 1–30) months.

A summary of the characteristics of all selected studies is provided in Supporting Tables S1 and S2.

## 4. Main Interventions or Comparators

### 4.1. Phase 2 Studies

Three primary studies compared belatacept alone with cyclosporine [6], any CNI [tacrolimus or cyclosporine] [15], or tacrolimus and MMF [16]. One study [12] compared combination regimens of belatacept [with MMF or sirolimus] with tacrolimus and MMF. For one study [6], the belatacept intervention arms were divided into MI and LI regimens. The dose of the intensive belatacept intervention arm was 10 mg/kg on Days 1, 5, 15, 29, 43, 57, 71, 85, 113, 141, and 169; then 5 mg/kg every 4–8 weeks for 7–12 months. The LI regimen used 10 mg/kg on Days 1, 15, 29, 57, 85, and 5 mg/kg every 4 or 8 weeks for 4–12 months, thereafter.

The only conversion study [15] used LI belatacept at 5 mg/kg IV on Days 1, 15, 29, 43, and 57, and then every 28 days thereafter. The study by Stock used rATG and belatacept at 10 mg/kg on Days 5, 14, 28, 56, and 84, then a maintenance dose of 5 mg/kg every 4 weeks [16]. Ferguson modified the belatacept dose to 10 mg/kg IV on Days 1 and 5, then once every 2 weeks through Month 3, every 4 weeks through Month 6, and 5 mg/kg every 4 weeks from Month 7 onwards [12].

### 4.2. Other Randomized Studies

Three primary randomized studies compared belatacept with cyclosporine [7, 8], or any CNI [20]. Similar to the Phase 2 studies, two [7, 8] used intensive (now, renamed MI and LI) doses of belatacept, as follows: 10 mg/kg q2 weeks over Months 0–3 and monthly from Months 4–6; then 5 mg/kg monthly thereafter; and 10 mg/kg q2 weeks in Month 1 and monthly over Months 2–3 then 5 mg/kg monthly thereafter. As the only conversion study, Budde et al. [20] used belatacept at 5 mg/kg IV on Days 1, 15, 29, 43 and 57, and then every 28 days thereafter. One study [19] compared monthly with every 2‐month dosing of belatacept (5 mg/kg monthly versus 5 mg/kg IV every 2 months).

Other studies used steroid‐sparing regimens [25, 26, 31]. In the CTOT‐10 study [26], there were 3 groups, as follows: Group 1 received alemtuzumab induction, rapid methylprednisolone taper, and maintenance tacrolimus and MMF. Group 2 received alemtuzumab induction, rapid methylprednisolone taper, and maintenance belatacept and MMF. For the control group, induction with basiliximab was given using a standard dosing regimen with rapid methylprednisolone taper, a 3‐month course of tacrolimus, and maintenance belatacept and MMF. The CTOT‐16 study [25] was similar, but rATG was used. Both studies were terminated early because of a high risk of thrombosis [26] and acute cellular rejection [25]. The study by Woodle [31] also used 3 groups—alemtuzumab/belatacept, rATG/belatacept, and a control arm of rATG/tacrolimus. Belatacept was administered at 10 mg/kg IV with the first belatacept dose given > 12 h and < 24 h after revascularization. The second belatacept dose was given between post‐transplant Days 4 and 6, and subsequent 10 mg/kg doses were given on Days 14, 28, 56, and 84. Thereafter, the belatacept dosage was reduced to 5 mg/kg given every 4 weeks through 24 months.

### 4.3. Nonrandomized Studies

Fifteen studies described comparator arms including tacrolimus [32, 33, 45, 49, 50], any CNI [34, 38, 39, 43, 48], or other immunosuppression combinations [35, 40, 51, 53, 54]. One study compared early versus late belatacept conversion [36].

A summary of interventions and comparators is provided in Supporting Table S1.

## 5. Risk of Bias in Included Studies

The risk of bias for Phase 2 and randomized studies is summarized in Supporting Figure S1.

## 6. Infectious Disease Outcomes

### 6.1. Phase 2 and Other Randomized Studies

#### 6.1.1. CMV (Figure 2).

##### 6.1.1.1. Belatacept Versus Calcineurin Inhibitors (Cyclosporine or Tacrolimus)

CMV infection in the short‐term (Analysis A 1.1.1; 6 studies, 2089 recipients; RR 1.25 [CI 0.86, 1.84]; *I* ^2^ = 3%) and medium‐term (Analysis A 1.1.2; 3 studies, 1098 recipients; RR 0.95 [CI 0.62, 1.44; *I* ^2^ = 0]) was equally likely in recipients receiving belatacept and those receiving CNI.

**FIGURE 2:**
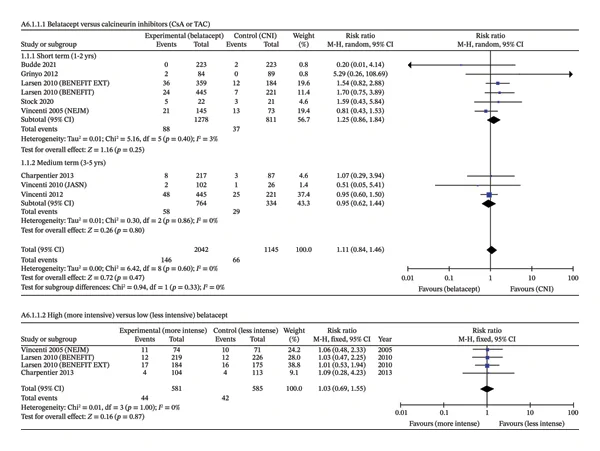
CMV.

##### 6.1.1.2. High (More Intensive) Versus Low (Less Intensive) Belatacept

Based on 4 studies (1166 recipients), CMV infection was equally likely among recipients who received MI and LI belatacept, with an RR of 1.03 [CI 0.69, 1.55, *I* ^2^ = 0] (Figure 2; Analysis A 6.1.1.2.).

Data from 2 long‐term studies were provided as CMV rates per 100 person‐years–2.2, 1.94, and 1.71 for MI, LI, and cyclosporine regimens in one [22], and 1.4, 1.1, and 0.8 in the other [30].

#### 6.1.2. PTLD (Figure 3).

##### 6.1.2.1. Belatacept Versus Calcineurin Inhibitors (Cyclosporine or Tacrolimus)

PTLD in the short term (Analysis B 1.2.1 ; 3 studies, 1207 recipients; RR 2.67 [CI 0.45, 15.82]; *I* ^2^ = 0%); medium term (Analysis B 1.2.2 ; 3 studies, 1122 recipients; RR 0.74 [CI 0.22, 2.51]; *I* ^2^ = 24%); and long term (Analysis B. 1.2.3 ; 2 studies, 1209 recipients, RR: 2.19 [CI 0.63‐7.62] ; *I* ^2^ = 0) was all equally likely in recipients receiving belatacept and those receiving CNI.

**FIGURE 3:**
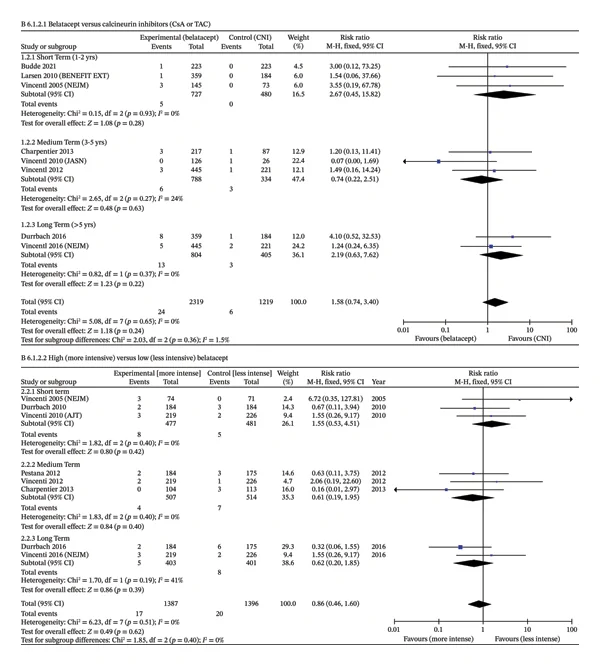
PTLD.

##### 6.1.2.2. High (More Intensive) Versus Low (Less Intensive) Belatacept

PTLD in the short‐term (Analysis B 2.2.1; 3 studies, 958 recipients; RR 1.55 [CI 0.53, 4.51]; *I* ^2^ = 0), medium‐term (Analysis B 2.2.2; 3 studies, 1021 recipients; RR 0.61 [CI 0.19, 1.95], *I* ^2^ = 0) and long‐term (Analysis B 2.2.3; 2 studies, 804 recipients; RR 0.62 [CI 0.20, 1.85], *I* ^2^ = 41%) were all equally likely in recipients receiving MI and LI belatacept.

#### 6.1.3. BK Infection (Figure 4).

##### 6.1.3.1. Belatacept Versus Calcineurin Inhibitors (Cyclosporine or Tacrolimus)

BK virus infection in the short‐term (Analysis C 1.3.1; 3 studies, 1285 recipients; RR 0.84 [CI 0.4–1.75]; *I* ^2^ = 0%) and medium‐term (Analysis C 1.3.2; 3 studies, 1098 recipients; RR 0.86 [CI 0.5, 1.47]; *I* ^2^ = 0) was equally likely in recipients receiving belatacept and those receiving CNI.

**FIGURE 4:**
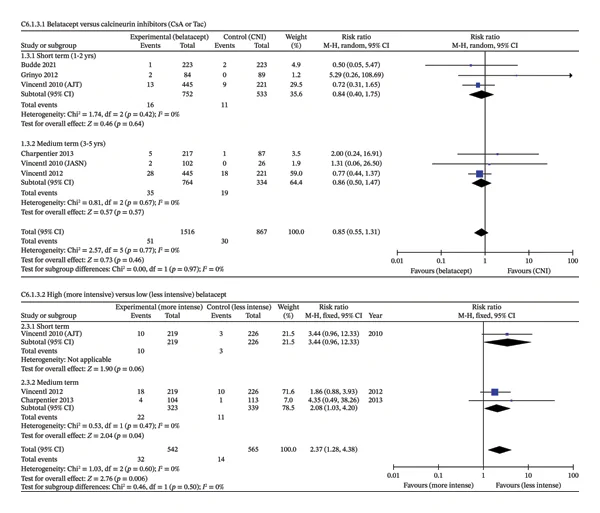
BK virus.

##### 6.1.3.2. High (More Intensive) Versus Low (Less Intensive) Belatacept

BK virus infection in the short‐term (Analysis C 2.3.1; 1 study, 445 recipients; RR 3.44 [CI 0.96, 12.33]) was equally likely in the MI and LI belatacept regimens. However, in medium‐term studies (Analysis C 2.3.2; 2 studies, 662 recipients; RR: 2.08 [1.03, 4.20], *I* ^2^ = 0), BK virus infection was twice as likely among those receiving MI versus LI belatacept dosing regimens.

### 6.2. Nonrandomized Studies

A total of 6 nonrandomized studies [32, 34, 38, 43, 48, 50] that evaluated belatacept versus CNI were analyzed. Based on these 6 studies with 1740 participants, CMV risk was two‐fold higher among those receiving belatacept versus CNI, with an RR of 2.15 [CI 1.58, 2.93, *I* ^2^ = 66%]. The risk of BK infection (1 study, 123 participants) was also greater than two‐fold higher, with an RR of 2.59 [CI 1.10–6.11]. However, the risk of PTLD (2 studies, 262 recipients) and BK PyVAN (1 study, 486 recipients) was equally likely for either belatacept or CNI, with RRs of 1.22 [0.19, 7.75] and 0.42 [0.15, 1.16], respectively. A summary of the analyses is provided in Supporting Table S3. The infection outcomes of cohort studies without comparators are summarized in Supporting Table S4.

### 6.3. Others

Studies evaluating other immunosuppressive regimens also evaluated infection outcomes, with no significant difference between the belatacept‐containing arm and the comparator (Supporting Table S5).

Two studies using registry data evaluated the risk of PTLD with belatacept versus CNI regimens. In one study [33], the risk of PTLD was not statistically significantly different, with an adjusted hazard ratio of 1.62 ([CI 0.89–2.95], *p* = 0.114). In the other [39], PTLD incidence was not significantly different at 5 years: 7.80 per 1000 patient‐years in the belatacept treatment group and 4.66 per 1000 patient‐years in the CNI treatment group (*p* = 0.18).

### 6.4. Case Reports

All case reports [60–65, 67–72] except for one [66] reported on solitary infections. The majority were viral infections, including CMV (*n* = 8), COVID‐19 (*n* = 1), hepatitis B (*n* = 1), JC virus (*n* = 1), HSV (*n* = 1), and VZV (*n* = 1); 2 were bacterial (*E. faecalis*, *Pseudomonas*, and *Enterococcus*), 1 parasitic (*Toxoplasma gondii*), and 1 fungal (*Pneumocystis jirovecii*). CMV disease occurred in 5 patients (1 colitis [66], 4 retinitis [63, 64, 68, 70]) and CMV DNAemia or viremia was reported in 3 patients [65, 66, 71]. Infections occurred a median of 25 (range 1.5–102) months after transplantation. Of those who converted to belatacept, infections occurred a median of 10 [1–11, 12–72] months after conversion. Belatacept was permanently discontinued in 6 patients [62, 65, 66, 68, 70, 72], but was resumed after temporarily being stopped in one [69]. Two of 13 patients died from overwhelming infection, and no allograft was reported lost. Supporting Table S2 summarizes the infections and outcomes of these reports.

## 7. Discussion

This systematic review and meta‐analysis of RCTs observed that the risk of CMV‐ and EBV‐associated PTLD was similar between kidney transplant recipients who received belatacept and those maintained on CNI, regardless of dose, in both short‐ and long‐term follow‐up studies (Supporting Table S6). While BK polyomavirus infection rates are similar between belatacept and CNI, the risk is higher among those receiving MI than LI belatacept during the 3‐ to 5‐year follow‐up period.

The safety profile of belatacept was initially re‐examined after early reports of high‐risk patients developing PTLD. In the initial phase 2 study of belatacept [6], three patients in the intensive belatacept arm developed PTLD, including two cases that occurred within 2–13 months after the discontinuation of the drug. Two of the three patients had primary EBV infections, while the third patient had also received lymphocyte‐depleting muromonab therapy. In the Phase 3 trial [8], a total of 6 patients developed PTLD, including 2 with central nervous system (CNS) PTLD; five of these patients received belatacept compared to only one in the cyclosporine cohort. Four of these patients had known PTLD risk factors. In an integrated safety profile analysis of belatacept [73], PTLD occurred more frequently among EBV‐seronegative patients, and more often in belatacept MI patients compared with LI or cyclosporine patients who received lymphocyte‐depleting therapy (MI: 5.7%; LI: 0%; cyclosporine: 0%) or who had CMV disease before the PTLD diagnosis (MI: 4.3%; LI: 1.5%; cyclosporine: 0%). With these findings, the U.S. FDA mandated that only EBV‐seropositive kidney transplant patients should receive belatacept. This mandate excluded the high‐risk populations in subsequent studies, which comprise the majority of studies in our review. Indeed, by restricting belatacept use to this non–high‐risk population, the risk of PTLD appears similar with other regimens, as seen in this meta‐analysis, and as reported in the population‐based studies [33, 39]. PTLD is a well‐recognized complication of transplantation with reported incidence rates of 0.3–1.4% in registries and large clinical series [74–76] and up to 2.9% in large, multicenter trials of immunosuppressive regimens [77]. The risk of PTLD is driven by EBV infection (especially during primary infection in immunocompromised transplant recipients with EBV donor‐positive/recipient‐negative mismatch status) and by the intensity (especially lymphocyte‐depleting) and duration of immunosuppression (which is augmented with the occurrence of acute cellular rejection).

Our analysis of RCTs demonstrates that the risk of CMV infection does not appear to be higher with belatacept use. In a safety analysis [73], CMV infection was not significantly different among belatacept‐ and cyclosporine‐treated patients (MI: 8%; LI: 7%; cyclosporine: 6%). Interestingly, this is in contrast to the result of 6 nonrandomized studies (1740 participants), in which CMV risk was two‐fold higher among those receiving belatacept versus CNI, with an RR of 2.15 (CI 1.58, 2.93, *I* ^2^ = 66%). The contrasting results may be due to differences in patient populations and the potential for unmeasured confounders, which are difficult to account for in non‐RCT settings. For example, 3 of 6 NRTs were conversion studies; this implies that the population at risk was previously exposed to other immunosuppressive regimens for varying periods, possibly indirectly contributing to a higher CMV risk. In addition, the dosing strategies for the underlying immunosuppressive regimens in these studies potentially differed, thereby impacting the overall risk of CMV infection. There are also other unaccounted confounders in NRTs that may have led to the observed increased CMV risk, including variable induction regimens, CMV D/R serologies at baseline, and the occurrence of acute cellular rejection. In addition, outcome misclassification is possible, as strict definitions for these infection outcomes were not described (e.g., cutoffs for CMV DNA levels may have varied; CMV disease and asymptomatic CMV infection were not considered separately). Regardless, our results imply that there may be heightened CMV vulnerability under costimulatory blockade, when used in real‐world settings outside of clinical trials, and thus, it would be important to implement measures for CMV surveillance and prevention [78].

During the medium‐term follow‐up of 3–5 years, the risk of BK virus infection and PyVAN was two‐fold higher with MI compared to LI belatacept. However, this observation must be interpreted cautiously, since the level of evidence is modest, based only on 2 studies, with a limited number of participants (*n* = 662). In general, the risk of BK virus infection, PyVAN, and graft failure is highest during the first 1–2 years after kidney transplantation. The higher dose of belatacept may cause an MI blockade of CD80 and CD86, resulting in reduced T‐cell surveillance or attenuation of virus‐specific CD8^+^ T‐cell responses. These results simply imply that there may be a need for continued BKV surveillance beyond the second year after kidney transplantation, among kidney recipients who are receiving the MI belatacept regimen.

Costimulatory blockade has also been implicated in other infections, including *Pneumocystis jirovecii* pneumonia (PJP), hepatitis B reactivation, and tuberculosis. Although we limited the meta‐analysis and review to the most common viral infections, the case reports highlight a range of other infections, from reactivation of other human herpes viruses (herpes simplex virus and varicella zoster virus), to toxoplasma, and PJP. However, it is important to point out that without control groups, belatacept alone cannot be implicated as the sole factor that increases the risk of these infections.

Our study has several limitations. First, we were unable to compare outcomes of CMV disease with asymptomatic CMV DNAemia since the majority of studies did not differentiate between these two conditions. As a result, outcome misclassification is possible, as strict definitions were also not specified for BK virus infections. Second, duplication across studies may have occurred, since cases from larger trials may have been described in detailed case reports, or outcomes may have been duplicated in long‐term follow‐up studies. Third, important abstracts or publications in non‐English journals may have been missed. Fourth, background immunosuppression was heterogeneous and varied substantially across studies, and this also likely contributed to the overall viral infection risk independently of belatacept. Finally, we limited the scope of the meta‐analysis to specific viruses such as CMV, EBV, and BK; other opportunistic infections were not assessed. We decided to focus on these outcomes because CMV and BK virus infections are the most common viral infections among kidney recipients, and earlier studies implied a higher risk of EBV‐PTLD with belatacept. Despite these limitations, this study is the first systematic review and meta‐analysis to focus solely on specific infectious disease outcomes.

## 8. Conclusions

Our systematic review and meta‐analysis of RCTs demonstrate that the risk of CMV does not appear to be increased by belatacept use. However, real‐world studies suggest a two‐fold higher risk of CMV during belatacept use. Among EBV‐seropositive kidney recipients, the risk of PTLD does not appear to be increased by the use of belatacept. In contrast, this meta‐analysis observed a potential for higher risk of BK virus infection, especially among patients receiving MI compared to LI belatacept. This implies a possible need to extend BK virus surveillance beyond the first 2 years after kidney transplantation to determine whether this translates to a subsequent increased risk for BK nephritis or allograft injury.

## Author Contributions

Cybele Lara R. Abad collected data; all authors contributed to data analysis, research design, discussion, and drafting of the manuscript.

## Funding

No funding was received for this manuscript.

## Disclosure

All authors have no relevant disclosures and approved the final version of the manuscript.

## Conflicts of Interest

The authors declare no conflicts of interest.

## Supporting Information

Additional supporting information can be found online in the Supporting Information section.

## Supporting information


**Supporting Information 1** File 1: Search strategy.

**Supporting Information 2** Figure S1: Risk of bias summary.

**Supporting Information 3** Table S1: Characteristics of included randomized and nonrandomized studies. Table S2: Characteristics of included case reports. Table S3: Infection outcomes of belatacept versus CNI‐containing regimens among nonrandomized studies. Table S4: Infection outcomes of cohort studies without comparators. Table S5: Infection outcomes of rATG + belatacept (*A*) versus rATG + TAC or MMF (B) containing regimens. Table S6: Summary of GRADE recommendations for randomized controlled trials.

## Data Availability

The data that support the findings of this study are available on request from the corresponding author. The data are not publicly available due to privacy or ethical restrictions.
