# Supplementary material for: Belatacept and the Risk of Cytomegalovirus, BK Polyomavirus, and Epstein–Barr Virus Post‐transplant Lymphoproliferative Disease After Kidney Transplantation: A Meta‐Analysis and Systematic Review
Source: J Transplant. 2026 Sep 12;2026:3256272. doi: 10.1155/joot/3256272 (PMC13570610; doi:10.1155/joot/3256272)
Supplement: Supplementary file 1 [file JOOT-2026-3256272-s001.docx]

| **STUDY AUTHOR, YR** | **SITE/COUNTRY/STUDY TYPE**  **(STUDY PERIOD)** | **N**  **(med ff up, mos)** | **Median Age, yr (range or SD) /(n, male)** | **STUDY ARM (N)** | **BELATACEPT DOSE** |
| --- | --- | --- | --- | --- | --- |
| **PHASE 2 RANDOMIZED TRIALS** | | | | | |
| **Vincenti 2005** | MC/multi-country  3/2001-12/2003 | 218 (12) | 46.5 /(73) | Belatacept MI (74) | 0 –3 Mo 10 mg/kg on days1, 5, 15, 29, 43, 57, 71, 85 4 – 6 Mo 10 mg/kg on days113, 141, 169 7–12 Mo 5 mg/kg every4 or 8 wk |
|  |  |  | 42.1 /(68) | Belatacept LI (71) | 0 –1 Mo 10 mg/kg on days 1, 15, 29 2–3 Mo 10 mg/kg on days 57, 85 4 –12 Mo 5 mg/kg every 4 or 8wk |
|  |  |  | 46.1 /(67) | CSA (73) |  |
| Vincenti 2010  5 yr ff up | MC/multi-country, LTE  3/2002-10/2007 | 138 (60) | ** | Belatacept (102) | ** |
|  |  |  |  | CSA (26) |  |
| Vincenti 2017  10 yr ff up | MC/multi-country, Post hoc  3/2002-10/2007 | (120) | ** | | 2nd randomization at 6 mos - 4 wk 5m/kg or 8 wk 5m/kg |
|  |  |  |  |  | 2nd randomization at 3 mos - 4 wk 5m/kg or 8 wk 5m/kg |
| **Rostaing 2011** | MC/multi-country  1/2007- NS | 173 (12) | 45.3 (13.5)/ (66) | Belatacept (84) | 5 mg/kg IV on days 1, 15, 29, 43, and 57, and then every 28 days thereafter. |
|  |  |  | 44.3 (13.0)/ (60) | CNI (89) |  |
| Grinyo 2012  2 yr ff up | MC/multi-country  SWITCH, LTE | (24) | ** | | |
| Grinyo 2017  3 yr ff up | MC/multi-country  SWITCH | (36) |  |  |  |
| **Stock 2020** | MC/US/open label  (2/2013-8/2015) | 60 (12) | 40 (29-53)/(12) | Bela + TAC +MMF (22) | 10 mg/kg on days 5, 14, 28, 56, and 84. After day 84, 5 mg/kg every 4 weeks |
|  |  |  | 39 (27-49)/ (13) | TAC + MMF (21) |  |
| **Ferguson 2011** | MC/multi-country/open label (7/2007-5/2009) | 89 (12) | 49.2 (11.1)/ (25) | Belatacept/ MMF (33) | 10 mg/kg IV on Days 1 and 5, then once every 2 weeks through Month 3, every 4 weeks through Month 6 and 5 mg/kg every 4 weeks from Month 7 onward. |
|  |  |  | 52.7 (10.8)/(20) | Belatacept/ SRL (26) |  |
|  |  |  | 53.6 (13.2)/ (22) | TAC, MMF (30) |  |
| **OTHER RANDOMIZED STUDIES** | | | | | |
| **Durrback, 2010**  **BENEFIT-EXT** | Phase III MC/multi-country (3/2005-NS) | 578 (12) | 56.7/(120) | Belatacept MI (184) | 10 mg/kg q2w over Months 0–3 and q4w over Months 4–6 5 mg/kg q4w thereafter |
|  |  |  | 56.1/(130) | Belatacept LI (175) | 10 mg/kg q2w in Month 1 and q4w over Months 2–3 5 mg/kg q4w thereafter |
|  |  |  | 55.7/(116) | CSA (184) |  |
| **Vincenti, 2010**  **BENEFIT** | Phase III, MC/multi-country (1/2006-6/2007) | 666 (12) | 43.6/(69) | Belatacept MI (219) | MI - 0-3 Mo 10 mg/kg Days 1, 5 Weeks 2,4,6,8,10, 12 4-6 Mo 10 mg/kg Weeks 16, 20, 24 7-12 Mo 5 mg/kg every 4 weeks |
|  |  |  | 42.6/(65) | Belatacept LI (226) | LI - 0-1 Mo 10 mg/kg Days 1, 5 Weeks 2, 4 2-3 Mo 10 mg/kg Weeks 8, 12 3-12 Mo 5 mg/kg every 4 weeks |
|  |  |  | 43.5/(75) | CSA (221) |  |
| Larsen 2010 | BENEFIT | (36) | ** | | |
| *2 yr ff up of Vincenti* | BENEFIT EXT | (36) |  |  |  |
| Pestana 2012  *3 yr ff up of Durrbach* | BENEFIT-EXT | (36) |  |  |  |
| Vincenti 2012  *3 yr ff up Vincenti* | BENEFIT |  |  |  |  |
| Charpentier 2013  *5 yr ff up Durrbach* | BENEFIT-EXT, LTE | 304 (5) |  | Belatacept MI (104) | ** |
|  |  |  |  | Belatacept LI (113) |  |
|  |  |  |  | CSA (87) |  |
| Rostaing 2013  *5 yr ff up Vincenti* | BENEFIT, LTE | 456 (5) |  | Belatacept MI (155) | ** |
|  |  |  |  | Belatacept LI (165) |  |
|  |  |  |  | CSA (136) |  |
| Durrback, 2016  7 yr ff up | BENEFIT EXT | (84) | ** | | |
| Vincenti 2016  7 yr ff up | BENEFIT | (84) |  |  |  |
| **Newell 2017** | MC/US/ CTOT-10  11/2011-NS | 19 (12) | 45 (30–63) / (4) | ALZ + MMF + TAC (6) |  |
|  |  |  | 40 (35–60) /(3) | ALZ + MMF + Belatacept (6) | 10 mg/kg on day 0 (day of transplant) and then on days 4, 14, 28, 56, and 84. Then maintenance dose of 5 mg/kg every 4 weeks until completion |
|  |  |  | 51 (37–63)/ (6) | BAS+TAC + MMF+ Belatacept (7) |  |
| **Mannon 2020** | MC/US/ CTOT-16  (8/2013-4/2015) | 69 (13) | 47 (34-62) /(20) | rATG+ MMF +TAC (29) |  |
|  |  |  | 46 (20-61)  /(21) | rATG+ MMF +Belatacept (29) | 10 mg/kg Days 1, 5, 14, 28, 56, 84 then 5mg/kg q4 wks until completion |
|  |  |  | 44 (23-59)/(7) | BAS + TAC + MMF +Belatacept (11) |  |
| **Woodle 2020** | MC/US  (9/2012-12/2016) | 316 (12) | 53.8 (43.2-60) /(30) | ALZ/Belatacept (107) | (10 mg/kg) given >12 hours and <24 hours after revascularization. The second belatacept dose (10 mg/kg) was given between PTDs 4 and 6, and subsequent 10 mg/kg doses were given on PTDs 14, 28, 56, and 84.  Belatacept dosage was reduced to 5 mg/kg given every 4 weeks out to 24 months. |
|  |  |  | 52.7 (45.35-59.8) / (38) | rATG/Belatacept (104) |  |
|  |  |  | 51.3 (42-63.2)/(36) | rATG/TAC (105) |  |
| **Badell 2021** | SC/US  (10/2015-8/2019) |  | 52/ (63) | Monthly Belatacept (82) | 5m/kg monthly |
|  |  |  | 50 /(54) | q2 Belatacept (81) | 5m/kg q 2 months |
| **Budde, 2021** | MC/multi-country  (4/2013-5/2017) |  | 53.4 /(150) | Belatacept (223) | 5mg/kg days 1, 15, 29, 43 and 57, and then every 28 days thereafter. |
|  |  |  | 52.6 /(151) | CNI (223) |  |
| Kaufman 2021  *2 yr ff up Woodle* |  | (24) | ** | | |
| **NON-RANDOMIZED STUDIES** | | | | | |
| Kirk 2014 | SC/US/PC  (NS) | 20 (12) | 45 (20–69)/ (12) | Belatacept + SIR  (DN) | 10 mg/kg postoperative day, repeated on days 3, 7 and 14, every 2 weeks for four additional doses, and monthly through month 6; thereafter belatacept was given at a dose of 5 mg/kg monthly |
| Cicora 2015 | SC/ARG/PC  (5/2013-12/2013) | 12 9 (NS) | 53.5 (27-73)/  (9) | ATG + Belatacept, MMA, CCS (DN) | ATG 2-5mmg/kg 5-7 doses; Bela 10mg/kg on PTD 1,5,15, 28,56, 84 then 5mkg q28 |
| Gupta 2015 | SC/US/RC (11/2012-12/2013) | 6 (9) | 40.2+/-8  (4) | Belatacept (CONV) | 5 mg/ kg on days 1, 15, 29, 42, 57 and then monthly |
| Brakemeier 2016 | MC/GER/CC (NS-11/2014) | 79 (NS) | 53.9±14.7  (48) | Belatacept vs. mTOR (CONV) | 5 mg/kg IV on days 1, 15, 29, 43, and 57 and then every 28 days |
| Le Meur 2016 | MC/FRA/RC (1/2012-2/2014) | 25 (20) | 59+/-13  (11) | Belatacept (CONV) | 5 mg/kg every 2 weeks for 2 mo and monthly thereafter. |
| Nair, 2017 | SC/US/RC (1/2012-12/2015) | 16 (18) | 61 (39-80)/  (14) | Belatacept (CONV) | <4 mos post transplant -- 10 mg/kg on days 1, 5, and every 2 weeks until 4 months post-transplant, then 5m/kg mo for maintenance;  >4 mos post-transplant -- 5 mg/kg on days 1, 15, 29, 43, 59, then 5 mg/kg every28 days |
| Schulte 2017 | SC/GER/RC  7/2012-5/2015 | 20 (12) | 52 +/-15/  (10) | Belatacept (CONV) |  |
| Wojciechowski 2017 | SC/US/RC  1/2012- 3/2015 | 20 (12) | 52.2 +/- 13/  (16) | Belatacept (CONV) | 10mg/kg Days 0,14,28 then 5m/kg days 42, 56 |
| Wojciechowski 2017 | SC/GER/RC  8/2012-7/2015 | 44 (12) | 56.9 (12.3)/  (26) | Belatacept (DN) | LI dosing on PTD 1, and second dose was PTD 7±3 |
| Darres 2018 | MC/EUR/RC (NS) | 219 (21) | 55.2 ± 13.8/  (155) | Belatacept (CONV) | NS |
| Schmitz 2020 | SC/US/RC  (5/2008-8/2014) | 40 (5) | 47 (20-69)/ (27) | Belatacept + SIR (DN) | 10 mg/kg on PTD 1 then day 3, 7, 14, every 2 weeks for 4 additional doses, and monthly through month 6. After that, reduced to 5 mg/kg monthly |
| Sethi 2020 | SC/US/RC  (7/2012-9/2017) | 108 (12) | 56 (44–64)/(71) | Belatacept (CONV) | 5 mg/kg administered intravenously every 4 weeks. |
| **NON RANDOMIZED STUDIES with COMPARATOR ARMS** | | | | | |
| Adams 2017 | Single/US/RC (5/2010-2/2015) | 758 | 51 /(57) | Belatcept (97) | 10 mg/kg during surgery and on PTD 4, 14, 28, 56, and 84, then (5 mg/kg) given every 4 wks thereafter |
|  |  |  | 55/ (64) | Belatacept/TAC short (87) | 10 mg/kg during surgery and on PTD 28, 56, and 84 + 8-12 ng/ml TAC; subsequent doses (5 mg/kg) given every 4 wks (wk 16, 20) + wean off TAC |
|  |  |  | 51/(56) | Belatacept/TAC extended (356) | as above then 4-24 mos 5mg/kg Belatacept q4 wks;  TAC wean 10-11 mos |
|  |  |  | 51/(60) | TAC (205) | 0-6 mos 8-12 ng/ml; 6-26 mos 5-8ng/ml |
| Leibler 2019 | Mult/FRA/CC (9/2014-9/2017) | 124 | 51+/-15 (34) | Belatacept (49) | 10 mg/kg at days 1 and 5 and at weeks 2, 4, 8, and 12 and then 5 mg/kg every 4 weeks. |
|  |  |  | 53+/-14  (56) | TAC or CSA/ MMF (74) | NS |
| Bertrand 2020 | Mult/FRA/RC (7/2010-7/2019) | 453 | 59.1 +/- 13 /(80) | Early (<6 mos) (121) | day 1, a second injection on day 15, a third on day 29, and then one injection every 4 weeks. |
|  |  |  | 50.2+/- 16/(205) | Late (>6 mos) (332) |  |
| Chavarot 2021 | Single/FRA/CC (3/2007-2/2019) | 262 | 59.2 (45.4-68.5)/(137) | Belatacept (181) | 5 mg/kg on days 0, 14, and 30, with subsequent  doses every 4 weeks |
|  |  |  |  | CNI (181) |  |
| Karadkhele 2021 | Single/US/RC  (1/2010-12/2017) | 308 | 48.0 [20.0, 77.0]/(216) | Belatacept (203) | 10 mg/kg at day 0 and weeks 4, 8, 12 followed by 5 mg/kg/ mo |
|  |  |  |  | TAC (105) |  |
| Magua 2022 | Single/US/CC  (1/2010-12/2017) | 173 | 52.1 (SD 11.7) /(73) | Belatacept (115) | 10 mg/kg at day 0 and at weeks 4, 8, 12 followed by 5 mg/kg monthly and a tapering dose of TAC targeting trough level of 5–8 ng/ml in the first 6 months, 3–5 ng/ml on month 6–9, and a subsequent wean-off phase to cessation by month 12 |
|  |  |  | 49.3 (13.2)/(29) | TAC (58) |  |
| Marvin 2022 | Single/US/CC  (1/2012-10/2020) | 328 | 52.3 (12.5) | Belatacept (164) | 5 mg/kg intravenously every 2 weeks for 6 doses, followed monthly thereafter. |
|  |  |  | 52.4 (12.1) | TAC (164) |  |
| Bertrand 2023 | Mult/FRA/RC (2007-2019) | 139 | 61.7 ± 11.1 (45) | Belatacept (69) | 5 mg/kg on days 1, 15, 29, 43 and 57 and then 28 days thereafter |
|  |  |  | 58 +/- 13.3 (41) | CNI (70) |  |
| Chiodo 2023 | Single/US/RC  (1/2015-9/2021) | 354 | 41.96/ (61) | Belatacept (119) | 5m/kg monthly w/o bolus |
|  |  |  | 38.25/ (115) | MMF/TAC/SRL  (235) |  |
| Moein 2023 | Single/US/CC  (1/2016-7/2022) | 96 | 53.89 ± 11.87/(32) | Belatacept (48) | Dependent on consultant |
|  |  |  | 54.52+/- 11.2/(32) | TAC/MMF/Pred(48) |  |
| Petrossian 2023 | Single/US/RC  (01/2015 to 10/2021) | 365 | 50.1/ (151) | TAC +SRL +MMF (246) |  |
|  |  |  | 51/(78) | Belatacept + MMF + TAC (119) | 5.0 mg/kg day 7 and day 30 post-transplantation and at 4-week intervals thereafter |
| Bertrand 2024 | Multiple/FRA/CC  (2011-2021) | 285 | 56.9 ± 13.9/(36) | Belatacept to CNI w/ TCMR (47) | 5 mg/kg intravenously on days 1, 15, 29, 43, 57 and then 28 days thereafter |
|  |  |  | 56.2 ± 14.8/(144) | Belatacept to CNI w/o TCMR (238) |  |
| Divard 2024 | Multiple/FRA/PCC  (5/2007-1/2020) | 486 | 52/(155) | Belatacept (243) | 5 mg/kg on days 0, 14, 28, 42, and 56 and then every 28 days |
|  |  |  | 51/(135) | CNI (243) |  |
| Schilsky 2024 | Single/US/RC  (1/2011- 12/2022 | 63 | 49.6 (SD 12.6)/(6) | CNI/MMF/  Belatacept (13) | 5mg/kg q 2 weeks x 5 doses then q monthly; dose adjustment w/ 10%change in BW; |
|  |  |  | 49.5 (SD 16.0)/(13) | Low dose CNI/Belatacept(22) | Dose taper of CNI or MMF by 50% by dose 2 then 25% by dose 3 then discontinue by dose 4 |
|  |  |  | 54.6 (SD 13.1) /(18) | MMF/  Belatacept (28) |  |
| Ahmed 2025 | Multiple/US/RC, propensity score matched  (6/2011-12/2019) | 126,678 | 57 (46-65)/(1006) | Belatacept (1514) | NS |
|  |  |  | 57 (45-65)/(5066) | TAC (7570) |  |
| Cherikh 2025 | Multiple/US/RC, propensity score matched  (6/2011-6/2016) | 61623 | 53.0 (42.0–63.0) /(1018) | Belatacept (1631) | NS |
|  |  |  | 53.0 (42.0–62.0)/(36319) | CNI (59992) |  |

**Table S1: Characteristics of Included Randomized and Non- Randomized Studies**

Studies in **bold** = Primary Studies; ** data as in primary study

**LEGEND**: ALZ – alemtuzumab, BAS – basiliximab, CC- case control, CCS – corticosteroids, , CNI- calcineurin inhibitor, CONV- conversion CSA – cyclosporine, CTOT - clinical trials in organ transplant, DN- de novo, EUR- Europe, FRA- France, GER- Germany, LI- less intense; LTE – long-term extension, MC- multi center, MI- more intense, MMA- mycophenolic acid, MMF- mycophenolate mofetil, mo- month/monthly, mTOR – mechanistic target of rapamycin, NS – not specified, PC- prospective cohort, Pred – prednisone, PTD- post transplant day, rATG – rabbit anti-thymoglobulin, RC- retrospective cohort, SC- single center, SD- standard deviation, SRL- sirolimus, TAC – tacrolimus, TCMR – T-cell mediated rejection

| **AUTHOR, YEAR,** | **AGE, SEX** | **DONOR** | **CMV**  **D/R** | **EBV** | **DN or CONV** | **TIME TO SYMPTOMS** | | **SYMPTOMS** | **INFECTION** | **TREATMENT / OUTCOME** | |
| --- | --- | --- | --- | --- | --- | --- | --- | --- | --- | --- | --- |
| Haidinger, 2009 | 64M | NS | P | P | DN | 57 | Acute kidney injury, diarrhea | | CMV disease | VGCV | Dead |
|  |  |  |  |  |  | 58.5 | Rising creatinine, LDH | | PJP | TMP/SFZ; BELA stopped |  |
|  |  |  |  |  |  |  |  | | *Enterococcus* | Teicoplanin |  |
|  |  |  |  |  |  |  |  | | *Pseudomonas* | TZP |  |
|  |  |  |  |  |  | 60 | Stomatitis | | CMV infection | Penciclovir |  |
|  |  |  |  |  |  |  |  | | HSV stomatitis | Ganciclovir |  |
| Cohen, 2016 | 50M | LR | P | P | DN | 1.5 | weakness, diarrhea, headache and poor appetite, with light headedness associated with hypotension. | | *E. faecalis* bacteremia | Daptomycin | Alive (18 mos) |
| Helou 2017 | 42F | LR | NS | P | CONV | 9 mos after 2nd ACR | painful dry tongue and sore throat | | Disseminated VZV | Acyclovir | Dead |
| Cambier 2019 | 47M | DD | NS | NS | DN | 25 | Elevated liver function tests | | Hepatitis B reactivation | Entecavir | Alive |
| Deliege2020 | 76M | DD (2nd) | P | P | CONV | 102 | decreased vision, right eye | | CMV retinitis | GCV>FOS(IV + intravitreal)+ MBV>FOS IV +CMV Ig | Alive |
| Marx 2020 | 58M | NS | NS | NS | CONV | 36 | Fever, cough | | COVID-19 | Supportive; BELA>CSA - then resume in 1 month | Alive |
| Smibert 2021 | 41M | DD | N/N | P | DN | 6 | Rectal pain, fatigue, dizziness, hematochezia, thrombocytopenia | | Primary CMV w/ immune thrombocytopenia | GCV+ IV Ig >VGCV | Alive |
| De Nattes 2022 | 53F | DD | N/N | P | CONV | 60 | Vertigo | | PML (also w/ 3 HZ recurrences) | Withdrawal of BELA + MMF | Alive (26 mos) |
| Fan 2022 | 41M | LR | NS | NS | CONV | 12 | Cloudy vision, floaters, right eye | | CMV retinitis | IV GCV + vitreal GCV + vitreal FOS | Alive |
|  | 52F | DD | NS | NS | CONV | 8 | BOV, both eyes | | CMV retinitis | IV GCV + vitreal FOS> VGCV+ vitreal FOS > IV + vitreal FOS | Alive |
| Kleiboker 2022 | 56M | DD | P/N | NS | CONV | 4 | Asymptomatic DNAemia / viremia  (40 738 IU/ml) | | CMV DNAemia / viremia | VGCV> Letermovir ; BELA stopped | Alive |
| Morel 2023 | 89F | NS | P | NS | CONV | 12 | blurring of vision, L eye | | CMV uveitis | VGV>GCV IV and vitreous; BELA stopped | Alive |
| Gani 2025 | 55M | DD | P/N | NS | CONV | 7 | Leukopenia,thrombocytopenia | | CMV DNAemia / viremia, persistent | MBV; BELA stopped | Alive |
| Vigilante 2025 | 71M | NS | NS | NS | CONV | 15 | Hyposthenia | | CNS Toxoplasma | TMP/SFZ; BELA stopped | Alive |

**Table S2: Characteristics of Included Case Reports**

**LEGEND:** ACR- acute cellular rejection, BELA- belatacept, CMV- cytomegalovirus, CONV- conversion, CSA- cyclosporine, DD- deceased donor, DN – de novo, F- female, FOS- foscarnet, GCV – ganciclovir, HSV- herpes simplex virus, HZ- herpes zoster, Ig – immunoglobulin, IV- intravenous, LDH- lactate dehydrogenase, LR- living related, M- male, mos – months, MBV- maribavir, N- negative, NS- not specified, P- positive, PJP -pneumocystis jirovecii pneumonia, PML- progressive multifocal leukoencephalopathy, TMP/SFZ – trimethoprim-sulfamethoxazole, TZP – piperacillin-tazobactam, VGCV- valgancivclovir, VZV- varicella zoster virus

| **Outcome or Subgroup** | **Studies,**  **N** | **Participants**  (# events/total participants Belatacept vs**.** CNI) | **RR [95% CI]** |
| --- | --- | --- | --- |
| CMV Infection | 6 | 1740 (97/803 vs 61/937) | 2.15 [1.58, 2.93] |
| PTLD | 2 | 262 (1/118 vs. 1/144) | 1.22 [0.19, 7.75] |
| BK Infection | 1 | 123 (12/48 vs. 7/ 74) | 2.59 [1.10, 6.11] |
| BK PyVAN | 1 | 486 (5/243 vs. 12/243) | 0.42 [0.15, 1.16] |

**Table S3: Infection Outcomes of Belatacept vs. CNI containing regimens among Non-Randomized Studies**

**LEGEND:** CMV- Cytomegalovirus, CNI- calcineurin inhibitor, PTLD – post transplant lymphoproliferative disorder, PyVAN- polyoma virus associated nephropathy

Footnote: Non-randomized studies (e.g retrospective) were pooled to evaluate primary outcomes -- CMV infection (e.g detection of CMV viremia or DNA in blood), PTLD, and BK infection (e.g. any BK virus viremia or DNA detected in the blood) or disease (e.g. PyVAN).

For CMV studies, 3 pooled studies were de novo belatacept use, while the other 3 were conversion studies.

| **STUDY AUTHOR YEAR** | **SERIOUS INFECTION** | **CMV (N)** | **BK** | **EBV** | **PTLD** | **Other** |
| --- | --- | --- | --- | --- | --- | --- |
| Kirk 2014 |  | Disease (1) | Viremia (10) |  |  |  |
| Cicora 2015 |  |  |  | 0 | 0 | *Pneumocytis jirovecii* pneumonia |
| Gupta 2015 | None |  |  |  |  |  |
| Brakemeier 2016 |  | Disease (1) |  |  | 1 |  |
| Le Meur 2016 | None | NS (3) | DNAemia / Viremia (2) |  |  |  |
| Nair 2017 |  | DNAemia / Viremia (3) | DNAemia / Viremia (1), Nephritis (1) |  |  |  |
| Schulte 2017 | None | DNAemia / Viremia (3) | DNAemia / Viremia (3), | 0 | 0 |  |
| Wojciechowski 2017 |  | Colitis (1) | DNAemia / Viremia (3) |  | 0 |  |
| Wojciechowski 2017 |  | DNAemia / Viremia (3), Disease (1) | DNAemia / Viremia (14), PyVAN (1) |  |  |  |
| Darres 2018 |  |  |  |  | 2 |  |
| Schmitz 2020 |  | DNAemia / Viremia (2) | DNAemia / Viremia (13), PyVAN (2) | DNAemia / Viremia (11) |  |  |
| Sethi 2020 | None | DNAemia / Viremia (8) | DNAemia / Viremia (5) | DNAemia / Viremia (15) | 0 |  |

**Table S4: Infection Outcomes of Cohort Studies Without Comparators**

**LEGEND:** NS- not specified, N = number, CMV- Cytomegalovirus, EBV – Epstein Barr Virus, PTLD – post transplant lymphoproliferative disorder, PyVAN- polyoma virus associated nephropathy

| **Outcome or Subgroup** | **Studies** | **Participants**  (# events/total participants A vs. B) | **RR [95% CI]** |
| --- | --- | --- | --- |
| CMV | 2 | 249 (18/133 vs 20/166) | 0.75 [0.4,1.41] |
| PTLD | 1 | 209 (1/104 vs 0/105) | 3.03 [0.12, 73.5] |
| BK DNAemia / Viremia | 2 | 267 (22/133 vs 12/134) | 1.82 [0.96, 3.45] |
| BK PyVAN | 2 | 240 (7/124 vs 2/116) | 2.5 [0.59, 10.57] |

**Table S5: Infection Outcomes of rATG+ Belatacept (A) vs. rATG + TAC or MMF (B) containing regimens**

**LEGEND:** CMV – cytomegalovirus, MMF- mycophenolate mofetil, PTLD – post transplant lymphoproliferative disorder, PyVAN- polyoma virus associated nephropathy, rATG – rabbit anti thymoglobulin, TAC -tacrolimus

| **EXPOSURE: Belatacept vs CNI** | | |
| --- | --- | --- |
| **OUTCOME** | **Grade, Strength of recommendation, Quality of evidence** | **Interpretation** |
| CMV Infection  Short/Medium term | 1B, Strong, Moderate | Use of belatacept does not increase risk of CMV infection or BK DNAemia or viremia in either short or medium-term studies, but evidence has important limitations (e.g., inconsistency, indirectness, imprecision). |
| BK Infection  Short term/ Medium term | 1B, Strong, Moderate |  |
| EBV-PTLD  Short term  Medium term/Long term | 1B, Strong, Moderate  2B, Weak, Moderate | Use of Belatacept does not increase risk of EBV-PTLD in short term studies, but evidence has important limitations  Use of belatacept in the medium to long term may have associated risk of EBV-PTLD, but there is uncertainty in effect estimates. Alternative management strategies may be reasonable. |
| **EXPOSURE: High vs Low Dose Belatacept** | | |
| CMV Infection | 1B, Strong, Moderate | Use of high dose belatacept does not increase risk of CMV infection but evidence has important limitations (e.g., inconsistency, indirectness, imprecision). Further research may influence confidence in the estimate of effect. |
| BK Infection  Short term  Medium term | 2B, Weak, Moderate  1B, Strong, Moderate | Use of high dose belatacept in the short term may increase risk of BK DNAemia or viremia, but there is uncertainty in effect estimates. Alternative management strategies may be reasonable.  Use of high dose belatacept increases risk of BK DNAemia or viremia in the medium term but evidence has important limitations (e.g., inconsistency, indirectness, imprecision). Further research may influence confidence in the estimate of effect. |
| EBV-PTLD  Short/ Medium/ Long term | 2B, Weak, Moderate | Use of high dose belatacept in the short/medium/ long term studies do not appear to increase risk of EBV-PTLD, but there is uncertainty in effect estimates. Alternative management strategies may be reasonable. |

**Table S6: Summary of GRADE Recommendations for Randomized Controlled Trials**
