# Supplementary material for: Belatacept and the Risk of Cytomegalovirus, BK Polyomavirus, and Epstein–Barr Virus Post‐transplant Lymphoproliferative Disease After Kidney Transplantation: A Meta‐Analysis and Systematic Review
Source: J Transplant. 2026 Sep 12;2026:3256272. doi: 10.1155/joot/3256272 (PMC13570610; doi:10.1155/joot/3256272)
Supplement: Supplementary file 3 [file JOOT-2026-3256272-s003.docx]

**Supplementary File : Search Strategy**

**A. Database: Ovid MEDLINE(R) 1946 to Present and Epub Ahead of Print, In-Process & Other Non-Indexed Citations and Ovid MEDLINE(R) Daily**
**Search Strategy:**
**1**  exp *kidney transplantation/ae (19713)
**2**  1 and (belatacept or abatacept).ti,ab,kw,mp. (155)
**3**  limit 2 to (english language and humans and yr="2005 -Current") (138)

B. **Database: Ovid MEDLINE(R) 1946 to Present and Epub Ahead of Print, In-Process & Other Non-Indexed Citations and Ovid MEDLINE(R) Daily**
**Search Strategy:**
**1**  exp Kidney Transplantation/ (110669)
**2**  exp lymphoproliferative disorders/ (412237)
**3**  2 or PTLD.ti,ab,kw. (413104)
**4**  3 or (infection* or virus* or viremia* or tuberculos*).mp. (3708707)
**5**  1 and 4 (20461)
**6**  exp Abatacept/ or belatacept*.ti,ab,kw. (3927)
**7**  5 and 6 (137)
**8**  limit 7 to (english language and humans and yr="2005 -Current") (126)

**Database: Embase <1988 to 2025 Week 47>**
**Search Strategy:**
**1**  exp *kidney transplantation/ (116369)
**2**  exp *lymphoproliferative disease/ (326686)
**3**  2 or PTLD.ti,ab,kw. (329412)
**4**  3 or (infection* or virus* or viremia* or tuberculos*).mp. (4385116)
**5**  1 and 4 (30391)
**6**  exp Abatacept/ or belatacept*.ti,ab,kw. (16355)
**7**  5 and 6 (493)
**8**  limit 7 to (human and english language and yr="2005 -Current") (459)
**9**  limit 8 to (adult or aged ) (310)
**10**  limit 9 to (clinical trial or randomized controlled trial or controlled clinical trial or multicenter study or phase 1 clinical trial or phase 2 clinical trial or phase 3 clinical trial or phase 4 clinical trial) (49)
