# Supplementary material for: Belatacept and the Risk of Cytomegalovirus, BK Polyomavirus, and Epstein–Barr Virus Post‐transplant Lymphoproliferative Disease After Kidney Transplantation: A Meta‐Analysis and Systematic Review
Source: J Transplant. 2026 Sep 12;2026:3256272. doi: 10.1155/joot/3256272 (PMC13570610; doi:10.1155/joot/3256272)
Supplement: Supplementary file 4 [file JOOT-2026-3256272-s002.docx]

**Supplementary Figure S1: Risk of Bias Summary**
